# Kidney stone disease: phenomenological perspectives

**DOI:** 10.1007/s11019-025-10301-7

**Published:** 2025-10-10

**Authors:** Chris A. Suijker, Corijn van Mazijk, Stijn Roemeling

**Affiliations:** 1 Department of Urology, University of Groningen, University Medical Center Groningen, Groningen, The Netherlands; 2 Faculty of Philosophy, University of Groningen, Groningen, The Netherlands

**Keywords:** Phenomenology, Lifeworld-led care, Kidney stone disease, Renal colic, Urolithiasis, Embodiment, Existentialism

## Abstract

Kidney stone disease is a highly prevalent condition, and has received significant attention in medical research due to its substantial impact on quality of life and the strain it places on healthcare systems. Despite its prominence, philosophical perspectives on kidney stone disease remain underexplored. This paper presents the first comprehensive phenomenological analysis of kidney stone disease, integrating both classical and contemporary phenomenological approaches with insights from qualitative and quantitative studies. We examine the lived experience of renal colic, the role of medical imaging, the diverse methods of treatment, and the challenges posed by recurrent chronic kidney stone disease, providing philosophical depth to these medical issues. We argue that kidney stone disease, along with its treatment, can deeply affect the patient’s relationship with their own body, sense of self, environment, and interpersonal connections. We conclude by discussing the practical implications of our phenomenological analysis for clinical care, advocating a more holistic, humanistic approach to kidney stone disease and its treatment. This approach recognizes not only the physical, but also the lived aspects of the condition, thereby enhancing patient care and well-being.

## Introduction

Kidney stone disease^1^ develops when urine in the kidney becomes supersaturated, leading to the mineralization of crystalloid and organic matter (Smith 2007). The condition has a multifactorial origin, influenced by environmental, hormonal, and genetic factors (Singh et al. 2022). It is one of the most common urological disorders, affecting 5–10% of the population in most industrialized countries (Sorokin et al. 2017). The incidence is rising, likely due to the impact of dietary habits and increasing body weight on urine solute concentrations (Meschi et al. 2011). Kidney stones vary in their location, composition, size, and rate of growth.

Although the presentation of kidney stone disease varies, ranging from asymptomatic cases to those with recurrent urinary tract infections, it is most commonly associated with renal (ureteral) colic, characterized by intense episodes of abdominal pain (Bultitude and Rees 2012). Due to its high prevalence, severity and tendency to recur—with up to 50% of patients developing new stones within five years—the disease significantly affects patients’ quality of life and places a substantial burden on healthcare systems (Khan et al. 2016; Hyams and Matlaga 2014). Management often requires repeated surgical interventions, dietary modifications, ongoing medical treatment, and regular follow-up care.

Contemporary understanding of kidney stone disease is predominantly rooted in the biological sciences. Advances in medicine, chemistry, and physics have led to the development of safe, minimally invasive surgical techniques and therapeutical strategies for preventing recurrence (Smith 2007). Like most somatic ailments, kidney stone disease is primarily approached from a third person, ‘natural’ perceptive, where the patients’ symptoms are interpreted through the epistemic authority of healthcare professionals and diagnostic techniques (Svenaeus 2023). In this framework, the disease becomes something that can be analyzed, explained, and treated.

These developments have contributed to the diminished role of patient experience in the clinical analysis of kidney stone disease, as well as in how consciousness and subjectivity shape the interpretation of this experience (Carel 2016, pp. 14–39). Today, patients with kidney stone disease are typically assessed in a detached, clinical manner by general practitioners, emergency physicians or urologists. Standardized protocols are followed: medications are prescribed, a computed tomography (CT) scan or other imaging is performed, and in some cases, a urinary stent is placed to relieve obstruction. Patients are often discharged to await spontaneous stone passage or scheduled for surgical intervention at the urology department.

Undoubtedly, modern medical advances have brought significant benefits in the management of kidney stone disease. Yet it is crucial to remember that it is primarily a lived and experienced phenomenon. Patients endure sudden and severe pain, often requiring surgical intervention, and when obstruction occurs, indwelling stents are inserted: devices that can significantly disrupt daily life (Corneli et al. 2023). For many, the disease is unpredictable and recurrent, taking on a chronic nature. The lived reality of these experiences cannot be captured by contemporary scientific analysis, which is primarily concerned with quantification and spatialization, often detached from the subjective dimensions of human experience. However, exploring the experiential aspects of kidney stone disease may lead to a deeper understanding of the condition itself and may enhance patient understanding and care (Carel 2011; Coninx and Stilwell 2021).

We therefore propose that the study of kidney stone disease could benefit from phenomenological analysis. Phenomenology is the systematic exploration of phenomena as they are consciously experienced, emphasizing the primacy of the first-person perspective (Smith 2018). As a complement to the natural sciences, phenomenology offers conceptual tools to better understand the essence of living with kidney stone disease and to reinterpret the broad array of changes it brings to a person’s life (Carel 2011). By foregrounding subjective experience, it acknowledges the lived burden of illness and opens up new possibilities for improving the therapeutic relationship between healthcare provider and patient (Toombs 1987; 1990; 2001). Central to the phenomenological method is the practice of ‘bracketing’ (*epoché*)—suspending assumptions and scientific frameworks—to focus on the experience and the individual’s comportment in the world (Smith 2018). Ultimately, phenomenology provides (self) understanding and may lead to a different conceptualization of pathologies, offering a more holistic view of illness.

The aim of this study is to explore and assess the lived experience of kidney stone disease. To achieve this, we will use prior phenomenological studies that examine the body and the lived experience of illness. To ensure the analysis remains grounded in actual patient experiences, we will incorporate findings from qualitative research, particularly semi-structured interviews with individuals who suffer from kidney stones. These accounts will serve to enrich and strengthen the phenomenological interpretation. Where appropriate, relevant quantitative data will also be considered, contributing to a dialogical phenomenological perspective that integrates both subjective and empirical dimensions (Geniusas 2022, pp. 11–40).

The article begins with a brief overview on the intersection of philosophy, phenomenology, and kidney stone disease, before moving into a detailed analysis of the condition. Special attention is given to the lived experience of renal colic and the recurrence of kidney stones. The discussion also explores the phenomenological impact of detecting (residual) stones through imaging, the experience of various interventions, and the role of dietary adjustments in prevention. Drawing on these analyses, the article concludes by outlining seven practical implications derived from the phenomenological inquiry.

## Philosophy, phenomenology, and kidney stone disease

Although the classification and conceptualization of kidney stone disease has a relationship to philosophy (Rapp 2014; Driggers 2023), and despite notable philosophers, such as Epicurus, Leibniz and Erasmus, suffering from the condition (O'Keefe 2014; Ananthaswamy 2019; Bisaccia et al. 2013), the relationship between philosophy and kidney stone disease has seen little development over time. While some qualitative studies have addressed the subjective perception of kidney stone disease through patient interviews, such accounts remain rare and are generally devoid of a deeper philosophical or phenomenological analysis.^2^ More broadly, philosophical engagement with illness itself has only gained momentum in the past century, as traditional philosophical thought often prioritized abstract contemplation over embodied, lived experience (Carel 2016, pp. 204–228).

Throughout the history of philosophy, illness has occasionally been granted philosophical meaning, but such reflections were typically isolated and never occupied a central position within the discipline. One notable exception is Michel de Montaigne (1533–1592), who famously asserted that the primary purpose of philosophy was to prepare us for illness and death (1580/1993). Perhaps not coincidentally, Montaigne himself suffered from severe kidney stone disease,^3^ which he described as “the worst disease, the most subtle, the most painful, the most mortal, beyond all remedy” (Bisaccia et al. 2013, p. 134). Nevertheless, it was not until the late nineteenth century that existence – and with it, illness – began to assume a more central role in philosophy.

It was during this period that Edmund Husserl (1859–1938) famously called for a return “to the things themselves” (1970/2001), marking the beginning of a new philosophical movement: phenomenology. This approach would later turn its attention to existence, perception, the lived body and eventually, illness (Smith 2018). Husserl’s early philosophy was primarily concerned with the first-person perspective, focusing on the cognitive structures and essential features of conscious experience. His successors expanded and deepened this framework: Martin Heidegger (1889–1976) shifted the focus toward the nature of human existence and *being-in the-world*; Maurice Merleau-Ponty (1908–1961) emphasized the primacy of embodied perception; Jean-Paul Sartre (1905–1980) explored themes of individuality, freedom and interpersonal relations; Simone de Beauvoir (1908–1986) applied phenomenology to explore the lived experience of women and the social construction of gender, extending phenomenology’s critical reach to issues of social inequality.

From the phenomenologist’s point of view, the natural sciences operate within a third-person framework, focusing on external objects of experiences—a perspective often referred to as the “natural attitude” (Geniusas 2022). In contrast, phenomenology suspends or ‘brackets’ such assumptions (*epoché*), setting aside scientific and everyday beliefs in order to investigate the essence of lived experience itself. Unlike approaches that study fixed objects, the “phenomenological attitude’” examines how phenomena appear to consciousness, exploring their subjective presentations and the cognitive structures that make such experiences possible. Crucially, phenomenology is not merely descriptive or limited to individual perspectives: it seeks an intersubjective understanding that reveals universal aspects of experience across individuals.

Although phenomenology, through methods such as bracketing, may appear to distance itself from the empirical sciences, it is better understood as complementary—particularly within the field of medicine (Carel 2011). Phenomenology offers a structured method for articulating the intersubjective essence of experience, enabling a deeper understanding of how health and illness are experienced and lived by patients. At the same time, phenomenology does not disregard scientific insights; rather, it often draws upon them, as disruptions in normal physiological functioning can illuminate how ordinarily taken-for-granted phenomena are structured. This dialogical reciprocity between phenomenology and science have facilitated its successful application across various domains of health care, including psychiatry, neurology, sexual medicine, and more recently, urology as well (Parnas and Zahavi 2002; De Preester 2013; Klaeson et al. 2012; Suijker et al. 2021).

## A phenomenological analysis of kidney stone disease

To undertake a phenomenological analysis of kidney stone disease, we begin by examining how phenomenology conceptualizes the body and its relation to the surrounding world. An understanding of these foundational concepts is essential for grasping how kidney stone disease affects not only the body, but also the person as a whole and their mode of *being-in-the-world.* In constructing a comprehensive phenomenological framework for kidney stone disease, we will draw upon both primary and secondary philosophical sources to examine a range of key experiences: the acute pain of renal colic, the diagnostic process through imaging, interventions such as stent placement and operative procedures, and preventive strategies. Finally, we will consider the phenomenological dimensions of living with recurrent kidney stone disease.

### The body, the person, and the world

Phenomenological analysis seeks to transcend the traditional divide between the subjective and the objective. Scientific approaches to the body are typically mechanistic, conceptualizing it as a system governed by physical and chemical processes. Within this framework, the body is treated as an ‘objective’ entity, while the mind is viewed as the ‘subjective’ locus where experiences are formed—both increasingly understood within a materialistic monism that seeks to reduce mental phenomena to biochemical mechanisms (Svenaeus 2000a). In this view, the mind is often positioned as the central structure responsible for shaping perception and controlling bodily action. However, despite its dominance, this materialistic model proves inadequate when attempting to understand the full experience of lived, embodied experience. Phenomenology offers an alternative by focusing on how the body is experienced from “within”, as both subject and object simultaneously.

In Husserl’s work, the body is not conceptualized merely as a physical object (*Körper*)—but as a lived-body (*Leib*) (Husserl 1989, pp. 151–169). This distinction is essential for analyzing bodily experience, as it acknowledges the body not as a detached object among other objects, but as a medium of perception with an “inner life” (*Innenleben*) that belongs to it (Husserl 1973, p. 269). The lived body is never simply “over there”, as other objects might be; it forms the ‘zero point’ of orientation (*Nullerscheinung*) for all experience (Geniusas 2022, pp. 120–141; Van Mazijk 2024). Moreover, the body is the very medium through which perception and interaction with the world occur. It is through the body’s sensory capacities that we apprehend and engage with our surroundings. As Merleau-Ponty describes, the body is not merely in the world, it “inhabits” it and “understands” it in a pre-reflective, practical way: it is our “general means of having a world” (Merleau-Ponty 1962/2012, pp. 139, 147). In this view, the body is not separable from human being^4^—it is pervaded by existence.

In our everyday activities, bodily awareness is typically not explicit, but pre-reflective (Merleau-Ponty 1962/2012, pp. 100–105). We carry out countless actions—walking to the supermarket, typing an article, gesturing in conversation—without consciously attending to our bodies. Most of the time, the body is taken for granted; we are accustomed to its seamless functioning and live with a sense of “bodily certainty” (Carel 2016, pp. 90). The body is not experienced as an external, inanimate object requiring directive control from the mind; rather, it is experienced as ourselves, as a unified and expressive whole (Merleau-Ponty 1962/2012, pp. 149–155). We pre-reflectively know the position of our limbs: we do not need to consciously objectify or locate them. Merleau-Ponty refers to this implicit bodily awareness and coordination as the body schema (1962/2012, pp. 100–105). While this unity is particularly evident in complex tasks like athletic performance, it is equally vital in seemingly simple actions, such as bending down to pick up a pen (Suijker et al. 2021). This understanding of the body stands in stark contrast to naturalistic conceptions found in scientific analysis.

In phenomenology, the nonobjective, unified lived-body, which typically operates pre-reflectively, is understood as fundamentally belonging to the person. According to Husserl, we can adopt a *personalistic attitude*, in which we experience the world from a first-person perspective that is bodily, mental, and spiritual (Moran 2009). Through this attitude, we engage with the world not as detached observers, but as persons who perceive, interpret and respond meaningfully. This stands in contrast to the *natural attitude*, in which human beings are primarily encountered unreflectively in everyday life or regarded as mere factual “things” among other things (Husserl 1989, p. 200). In the personalistic attitude, we are also situated within a particular social and historical context that shapes how we conduct ourselves and how we make sense of our experiences. At the same time, we are active agents in the creation of meaning: meanings that contribute to and help structure the shared social and the historical world. The person, then, is not merely a biological or a psychological entity, but an “embodied, self-conscious, social, historical, and engaged self” (Geniusas 2022, p. 146).

As Merleau-Ponty writes, “the life of consciousness […] is underpinned by an ‘intentional arc’ that projects around us our past, our future, our human milieu, our physical situation, our ideological situation, and our moral situation, or rather, that ensures that we are situated within all these relationships” (1962/2012, p. 137). This *intentional arc* lies at the heart of personhood, expressing how lived experience is shaped by and accumulates within our own personal history. It is through the intentional arc that our existence becomes coherent, rooted in past experiences and oriented towards future possibilities (Merleau-Ponty 1962/2012, pp. 136–139). The intentional arc allows for continuity and meaningful engagement with the world; it enables us to act purposefully, interpret events through our lived context, and remain connected to the social, physical, and moral dimensions of our surroundings. In this way, existence is not detached, but situated and meaningful.

In addition to the body and the person, the world itself requires phenomenological interpretation. Unlike the natural sciences, which tend to view the world as an external, objective reality to be measured, categorized and rationally understood, phenomenology conceives of the world as a *life-world (Lebenswelt)* (Husserl 1970; Svenaeus 2000b) The world is the space in which we live, act, and find meaning; it forms the ever-present horizon of all our activities. Crucially, the *life-world* is not passive or neutral—it continually invites us to act, enabling us to pursue and sustain or life projects. As Husserl states, our fundamental mode of being in the world is not captured by the statement “I think that”, but rather by the embodied “I can” (1973, p. 46).

Invoking Heidegger’s phenomenology, this *being-in-the-world* is also marked by a sense of being at home in the world (Heidegger 1962/2001, p. 233). Normally, we are attuned to our surroundings, responding fluidly and confidently to the situations we encounter. In this way, we are not merely placed in the world “as a thing among other things”, but are fundamentally integrated with it (Svenaeus 2011, pp. 336). Furthermore, Heidegger emphasizes the intersubjective nature of existence (*Mitsein* or *Mitdasein*): our being is always already shaped by the presence of others (Heidegger 1962/2001, pp. 153–163). The world is saturated with signs of human activity, and our orientation toward the world is structured by shared norms, goals and expectations.

Drawing on phenomenology, we established that (i) the body is pre-reflective, non-objective, unified, and lived; (ii) the person is shaped by the personalistic attitude and sustained by the intentional arc; (iii) the world is the intersubjective, homelike horizon of meaningful action. In what follows, we will explore how the symptoms, diagnostic procedures, and management strategies associated with kidney stone disease disrupt the patient’s experience of the body, sense of self and relationship to the world.

### Symptoms: renal colic

Kidney stone disease is most notably characterized by renal (ureteral) colic. Medically, renal colic is defined as “the pain arising from obstruction of the ureter […], *which* is caused by spasm of the ureter around the stone, causing obstruction and distention of the ureter, pelvicalyceal system, and renal capsule” (Bultitude and Rees 2012). Clinically, this manifests as severe intermittent pain typically originating in the flank (loin) and radiating toward the back, the groin or genital region, depending on the stone’s location. The pain is often acute and disabling, and is frequently accompanied by a range of other symptoms, including lower urinary tract symptoms (such as frequency, urgency, or dysuria), nausea, vomiting, and restlessness.

Patients experiencing renal colic are typically assessed by general practitioners, emergency physicians or urologists in acute care settings (Bultitude and Rees 2012). Standard medical management includes the administration of analgesics and symptom-relieving medications, followed by diagnostic imaging to identify the stone. In cases where pain persists despite medical therapy, or in cases of severe obstruction, interventional measures may be taken, such as the insertion of a ureteric stent or percutaneous nephrostomy to alleviate obstruction. In approximately 10–20% of cases, stones fail to pass spontaneously, and minimally invasive surgery is scheduled several weeks after the first episode (Auge and Preminger 2002). Following resolution, patients may receive dietary advice and pharmacological measures aimed at preventing recurrence, a process referred to as metaphylaxis.

The literature frequently refers to renal colic as “one of the most severe physical pains a human can experience” (Ní Néill et al. 2023, p. 706). This is echoed in qualitative studies, where patients describe the intensity of the pain in striking terms, including reports of suicidal ideation and the wish to “remove all the organs to be relieved” (Ayyad and Ayaad 2023, p. 5; Ní Néill et al. 2023). Drawing on prior phenomenological work and the foundational concepts outlined in the preceding section, we propose that acute renal colic disrupts experience in at least four ways: (i) it forcibly draws attention to the body, (ii) it objectifies the body, (iii) it temporarily suspends our ability to pursue projects in the world, and (iv) it inscribes itself into the intentional arc, shaping future bodily awareness. As will become clear in subsequent sections, these phenomenological effects differ from those associated with recurrent or chronic kidney stone disease, which demand a separate analysis.

As our phenomenological introduction has shown, in ordinary day-to-day interactions, the body is primarily lived and subjective. It operates pre-reflectively, providing a stable, taken-for-granted basis for our actions. In this mode, the body recedes into the background of experience: it is not the object of our attention. As Gallagher and Zahavi put it, “it tends to efface itself on its way to its intentional goal” (2008, p. 143). The body enables action rather than obstructing it. However, in the case of renal colic, this foundational certainty is radically disrupted. The pain does not emerge as a symptom to be endured: it imposes itself, invading consciousness and demanding attention.

In his analysis of Husserl’s work, Saulius Geniusas writes that “pain originates as a rupture in the field of experience that unsettles one’s otherwise natural absorption in the world of things” (2022, p. 38). Pain, in this sense, forces the body out of its usual transparency and into the foreground of experience (Coninx and Stilwell 2021). This is especially true for renal colic. Under normal conditions, we remain immersed in our everyday projects, largely unaware of our body. But when an episode of renal colic strikes, this absorption is abruptly shattered. The body becomes unpredictable: it moves restlessly, it becomes nauseated, and consciousness is consumed by pain. All sense of habitual fluency collapses. The body seems to acquire a will of its own, as Toombs describes (1987), and the patient becomes, in Geniusas’ words, “disconnected from the painless past and the painless future” (2022, p. 111). As one patient describes: “the pain is uncontrolled, you cannot do anything during the pain attack that came suddenly almost every day” (Ayyad and Ayaad 2023, p. 5).

Phenomenologists further argue that the body in pain takes on a more objective character (Carel 2016, pp. 40–64). Pain is often localized—it is felt in a particular bodily region – and this focused attention fragments the usual sense of bodily unity and wholeness, as consciousness isolates the painful part and seeks to understand the meaning of pain or how to alleviate it. In this way, pain inherently objectifies the body (Svenaeus 2015; Sartre 1957/2003, pp. 378–380). Rather than being absorbed in the world around us, pain forces us to confront our body. As Geniusas states, “pain freezes the body, objectifies it, and distances it from the subject of experience” (2022, p. 138). As we will explore in later sections, medical imaging and clinical encounters often reinforce this objectification by deepening the analysis of the causes and cures of pain, while also shifting attention from raw lived experience to technical or numerical analysis, further disrupting the body’s experiential unity.

A third aspect of our analysis of renal colic concerns the patient’s diminished ability to maintain or create future-oriented projects. During a period of intense pain, attention is fully absorbed by the body, rendering the sufferer incapable of engaging with the *life-world* or sustaining meaningful connections with others (Carel 2016, pp. 65–85). The sudden onset of renal colic interrupts daily routines and abolishes personal plans, resulting in a temporal disconnection from both the pain-free past and the painless future (Geniusas, pp. 97–119; Toombs 1990). As Toombs observes, “the future is suddenly disabled, rendered impotent and inaccessible” (1987, p. 234). The individual range of possible actions becomes severely restricted; all that remains is the urgent pursuit of pain relief. Renal colic thus entails the loss a previously familiar life-world, one in which various physical, social and existential activities were taken for granted. This disruption is supported by qualitative and quantitative studies on kidney stone disease, in which patients report significant limitations in work, travel, social interaction, sexual activity, and other daily or weekly tasks (Ní Néill et al. 2023; Ayyad and Ayaad 2023; de Bayser et al. 2023; Ragab et al 2020).

The final dimension of our analysis concerns the intentional arc. As discussed earlier, the body carries a kind of pre-reflective memory: past experiences shape how we respond to future situations (Merleau-Ponty 1962/2012, pp. 137–139). A renal colic attack, due to its severity and disruptiveness, often leaves a lasting impression on the intentional arc. The experience does not remain isolated in time, but it influences the individual’s future bodily awareness and reactions. One patient, reflecting on their experience, explains: “I am worried, like as soon as I start getting like a little pain in my stomach, I am panicking” (Raja, Wood & Joshi 2020, p. 231). Another notes: “If something does not feel right for a second or two you are like, oh no – I’m going to have to think about talking to my boss about potentially being out of a day or two to get surgery done” (Ní Néill et al. 2023, p. 714). These testimonies illustrate how renal colic is not a past event, but how it influences the interpretation of future bodily signals. Other studies similarly highlight the ongoing anxiety, vigilance, and stress that stem from the fear of recurrence (Ayyad and Ayaad 2023; de Bayser et al. 2023; Ragab et al. 2020), demonstrating how the intentional arc is reshaped after a renal colic attack.

Naturally, these experiential disruptions are most intense during acute renal colic. After the pain subsides, the changes it provokes in the experience of the body and world can—at least partially—recede or “recover” (Carel 2016, p. 93). As one patient puts it: “and then they [kidney stones] are gone and off you go again” (Ní Néill et al. 2023). Whether the episode leaves lasting phenomenological traces on the body, world and sense of self, is perhaps most dependent on the chronicity of the condition (Coninx and Stilwell 2021). Chronic or recurrent kidney stone disease introduces a different set of experiential structures than a single, isolated renal colic episode—ones we will return to in due course. For now, however, we turn our attention to the phenomenological experience of medical imaging in kidney stone disease, where the dynamic between subjectivity and objectivity becomes particularly striking.

### Diagnosis: localizing disease

The idea that pain can lead to an objectifying shift in the lived body has already been introduced. However, in kidney stone disease, there is a second way in which the body becomes objectified. Patients suspected of having kidney stones typically undergo abdominal radiographs, ultrasonography, or more commonly, abdominal CT scans to visualize the stones and determine their location (Brisbane et al. 2016). While these diagnostic tools assist physicians in determining treatment, they also bring about significant changes in how patients perceive their body. By localizing the stones within a specific anatomical structure through imaging, patients begin to visualize their bodies more as objects (*Körper*), which alters the lived experience of the body (*Leib*) (Svenaeus 2023).

Through imaging, kidney stone disease transitions from something to be experienced to something to be observed (Toombs 1990). As the scan is examined, the body is no longer perceived as fully belonging to the patient; instead, it becomes an object. Toombs argues, “with objectification comes alienation. As an object, the body is suddenly perceived as a ‘thing’ which is exterior to the self, as something Other-than-me” (1988, p. 214). Furthermore, the language used by physicians often reinforces this objectification: kidney stone disease is described as something localized within the body, with a mechanistic cause and a technological solution. Sartre notes that through diagnosis, illness transforms into disease, becoming “objectively discernible for Others” (1957/2003, pp. 378–380).

Therefore, in both clinical encounters and beyond, patients are constantly required to shift perspective: from experiencing the body from within to observing it from the viewpoint of the ‘Other’ (Carel 2016, pp. 40–64). Some phenomenologists liken this process to two hands touching each other: the body is both touched and touching at the same time—one moment it is subject, the next it becomes an object (Merleau-Ponty 1962/2012, p. 95). This continuous shift can be exhausting and, at times, lead to a sense of alienation (Toombs 2001). However, rather than a simple back-and-forth switching of perspectives, viewpoints of others can also blend or integrate. The lived experience of pain may be significantly shaped by the seriousness of the diagnosis or the meanings attributed to it by others. Thus, the experience of pain is influenced not only by shifting perspectives, but also by the social and interpretive context in which it occurs.

In addition to the objectification of the body, there is another way in which imaging influences the patient’s perception and experience. After treatment, imaging is often conducted to identify any residual stones. Although larger fragments are typically removed, smaller ones are frequently left behind (Chung et al. 2019). This finding is discussed by doctor and patient, which can contribute to changes in the lived experience of the body and the way the person relates to it. When residual fragments are detected, patients may feel that their body has not been sufficiently healed, and this can foster a sense of chronicity and uncertainty about the future. One patient reflects, “In my opinion, I have not yet got well because there are still leftover kidney stones” (Nouri, Hassali & Hamzah 2021). Others express similar concerns, such as: “it’s [kidney stone disease] kind of a ticking time bomb […] you just do not know when it is going to react” (Ní Néill et al. 2023, p. 712; Raja, Wood & Joshi 2020).

This also demonstrates how the experience becomes embedded in the intentional arc. One study notes that patients with residual stones sometimes refrain from making plans or travelling due to anxiety about potential recurrence (de Bayser et al. 2023). It is important to acknowledge, however, that similar feelings can arise in patients who are fully stone-free, as new stone formation occurs frequently even without residual fragments. Nonetheless, we argue that those with residual fragments may have a more challenging ‘starting point’. The presence of stones, even small, directly objectifies the body after treatment, fostering a sense of uncertainty about future episodes.

Physicians may eventually offer solutions to the challenges of objectification and the impact that imaging has on our perception on the future. However, before suggesting any practical approaches, the phenomenological exploration must be completed. The management of kidney stone disease and recurrent chronic cases still requires a thorough phenomenological interpretation to fully understand its effect on the patient’s experience.

### Management: stents

Kidney stone disease can be managed in several ways, including conservative treatments, extracorporeal shock wave lithotripsy (ESWL), or minimally invasive renal surgery, either antegrade or retrograde (Auge and Preminger 2002). In some cases, a urinary stent is placed to temporarily relieve obstruction before spontaneous stone expulsion or surgery (Beysens and Tailly 2018). These stents can be placed in different locations: internally between the bladder and the kidney (double-J stent) or externally between the skin of the loin and the kidney (nephrostomy stent) (Weltings et al. 2019). Stents are often placed during surgery as well, where they are left indwelling for a few days up until a few weeks to prevent complications such as ureteral swelling and blood clot obstruction (Srinivisan et al. 2009).

Although ureteral stents are generally effective in relieving the symptoms of obstruction and are often better tolerated than renal colic pain, some patients experience significant discomfort from the stent itself. One patient describes it as “physically nauseating, overwhelming pain”, while another recounts having “crawled home, got in bed, and stayed there until [she] went back to get the stent out” (Corneli et al 2023, pp. 645–646). Yet another states it was “the most horrible experience” (Nouri, Hassali & Hamzah 2021). For these patients, we argue that the stents can evoke similar phenomenological effects as to those experienced during renal colic. The presence of an indwelling stent that causes discomfort shifts the body from being a pre-reflective lived-body, to an objectified *Körper* that moves to the foreground. The stent’s discomfort thwarts future projects, rendering the individual unable to engage in the life-world as before. One patient expresses this feeling: “I felt lousy, terrible […] I was disintegrating, fading from the face of the world” (Kelly and Kelly 2019, p. 34), while another one shares, “My entire week was dictated by the stent” (Corneli et al. 2023, p. 646).

There is less qualitative research on the experience of external nephrostomy stents, but it is known that these stents typically result in fewer urinary tract complaints, but their placement is still painful, and some patients continue to experience significant discomfort afterwards (Bigum et al. 2015). From a phenomenological perspective, we argue that these nephrostomy stents contribute to a shift from the lived-body to the *Körper*: a tube is physically felt exiting the body through the loin, and the patient must carry a urinary bag. The nephrostomy stent and its accompanying bag must be integrated into the body schema, as being unaware of the tube’s position can lead to complications, such as the stent becoming stuck, hooked or compressed, causing obstruction, pain, leakage, or dislocation. The stent also disrupts future projects: exercising becomes difficult, as does intimacy, or sleeping on the side with the tube (Bigum et al. 2015). Additionally, any issues like displacement, leakage, or obstruction necessitate (emergency) replacement, further constraining the patient’s future actions (Joshi et al. 2001).

Both the experiences with internal stents and nephrostomy stents become embedded in the *intentional arc*: the pre-reflective memory of past experiences which shapes how we respond to future situations. In a study, several patients expressed a preference for receiving the most effective treatment, even if it was more invasive, in order to avoid enduring time with internal stents (de Bayser et al. 2023). This aligns with our clinical experience, where patients who have previously suffered from stent-related discomfort tend to prefer more invasive treatments to avoid further stent replacements, even if these come at higher costs. Additionally, patients experience anticipatory anxiety about reinterventions, especially since these may involve the placement of an internal stent (de Bayser et al. 2023). Stents may also trigger fear of infection or malfunction, further influencing future treatment decisions (Bigum et al. 2015).

### Management: extracorporeal lithotripsy and surgery

Interventions aimed at stone fragmentation include ESWL and minimally invasive surgery, such as ureterorenoscopy and percutaneous nephrolithotomy. While surgery is normally performed under general anaesthesia, ESWL is not (Auge and Preminger 2002). In ESWL, shock waves are targeted at the body with the aim of fragmenting the stones, so they can later pass spontaneously. These shockwaves can be painful (Mahmood et al. 1998). During the procedure, patient must lie still, surrounded by the shock wave generator and the X-ray equipment used to locate the stone. This setting can contribute to the objectification processes of the body, disrupting the experience of the body as lived. On the X-ray screen, the body becomes a visual object, and the patient is acutely aware that the treatment targets a specific anatomical site. In this moment, the body is no longer a unified, pre-reflective lived body, but becomes something acted upon—a passive object. This shift may evoke feelings of vulnerability and a loss of agency, as the patient must remain motionless while the body is subjected to the external intervention.

Additionally, the experience of temporality may significantly be altered during this procedure. In his analysis of Husserl’s interpretation of temporality in relation to pain, Geniusas argues that pain generates a “field of stagnating presence, a time that come to a standstill” (Geniusas 2022, p. 111). It is crucial to distinguish between objectively measured time and subjectively experienced time. While this temporal disruption is evident in renal colic, it becomes even more pronounced during ESWL, where the patient remains conscious throughout the treatment. For the treating physician or technician, the procedure might last only 30–45 min (Yilmaz et al. 2005). However, for the patient—required to remain immobile while receiving shockwaves—time is not experienced as linear or neutral. Discomfort or pain draws attention to the present, dissolving meaningful connections to the past or future (Geniusas 2022, pp. 97–119). In fact, even a relatively short duration, such as 30 min, may subjectively feel much longer for the patient. As a result, time becomes a deeply personal and isolated experience, markedly different from that of the clinician, emphasizing the disjunction between the lived-body and its treatment.

Minimally invasive surgery under general anaesthesia is experienced in a fundamentally different way. Due to the suspension of consciousness, the patient’s embodied awareness and their relationship with the world are temporarily put on hold. This interruption, although it may be experienced positively in many cases, can also provoke feelings of bodily vulnerability, depersonalisation, and alienation, as the body is rendered passive and subject to the actions of others (Sheen and Oates 2005). These experiences correspond with empirical findings in which patients express anxiety about not waking up from surgery, highlighting deep concerns around loss of control and bodily autonomy (Mavridou et al. 2013). Upon waking, patients frequently encounter a temporal gap: they struggle to piece together what occurred during the period of unconsciousness and how they are situated within their temporal horizon. Furthermore, qualitative research indicates that patients often become preoccupied with the presence of medical tubes postoperatively (Mavridou et al. 2013; Worster and Holmes 2009). This observation resonates with our earlier phenomenological discussion of stents, reaffirming their disruptive impact on lived experience and the body schema.

### Management: metaphylaxis

The final component in the management of kidney stone disease is metaphylaxis, or the secondary prevention of new stone formation. Metaphylaxis involves both general and specific measures, which are tailored based on individual risk factors and, when available, the type of stone identified after extraction or spontaneous expulsion (Skolarikos et al. 2024) General preventive strategies include maintaining a daily fluid intake of 2.5–3 L, engaging in regular physical activity, and following a balanced diet rich in vegetables and fibre, with normal levels of calcium while limiting salt, animal protein, alcohol and carbonated beverages. When stone composition or laboratory results reveal specific metabolic abnormalities, additional pharmacological treatments may be indicated. These may involve medications that alter urinary pH or correct electrolyte imbalances to reduce the risk of recurrence (Skolarikos et al. 2024).

This shift in management introduces a phenomenological loss of spontaneity. As previously discussed, the body is typically experienced as a pre-reflective, lived-body. However, the imposition of dietary and behavioral constraints alters this mode of being. For patients with kidney stone disease, especially those who struggle to maintain adequate hydration or need to avoid specific dietary components, formerly automatic behaviors now require planning and self-monitoring (Streeper et al. 2019; Carel 2016, pp. 73–74). The body no longer simply cooperates with the patient’s desires; instead, it becomes a site of regulation and control. This necessity for active intervention transforms ordinary actions and objects—such as a glass of water or a meal—into tools of compliance or sources of risk, making it ‘useful’ or ‘useless’. As a result, the body shifts from being a lived, taken-for-granted medium of engagement with the world to an objectified entity requiring conscious oversight. This phenomenon may be particularly evident in patients with uric acid stones, who may need to closely monitor and regulate their urinary pH through medication in order to dissolve the stones non-surgically (Shekarriz and Stoller 2002).

## Recurrent kidney stone disease

So far, we have examined the phenomenological implications of renal colic, diagnostic processes, and the various forms of treatment for kidney stone disease. For many patients, these experiences are temporary, and accordingly, the phenomenological disruptions they cause—such as the shift from a pre-reflective lived-body to a fragmented, objectified body; the inability to sustain future-oriented projects; and the associated feelings of alienation and depersonalization—tend to recede once the acute phase resolves. Over time, these disruptions may become encapsulated in the past, and their grip on daily life begins to loosen. However, for a significant subset of patients, kidney stone disease becomes a chronic condition. Empirical studies suggest a recurrence rate of up to 50% within five years (Khan et al. 2016). Some individuals experience frequent relapses or develop complex stones that require multiple surgeries. In particularly severe or refractory cases, even radical measures such as surgical removal of an entire kidney are considered (Carvalho et al. 2013).

We argue that the recurrent nature of kidney stone disease—prompting some researchers to propose its reconceptualization as a chronic condition characterized by episodic exacerbations (de Bayser et al. 2023)—produces a fundamentally different phenomenological impact compared to its transient manifestation. Chronic illnesses, particularly those involving recurrent pain such as renal colic, engender more enduring disruptions in one’s lived experience (Coninx 2024). In the context of chronic kidney stone disease, at least five key phenomenological dimensions emerge: (i) bodily doubt, (ii) a ‘limp’ intentional arc, (iii) depersonalization, (iv) a tendency of psychologization and somatization, and (v) an experience of unhomelike *being-in-the-world*.

The notion of bodily certainty, where the body functions as the transparent medium through which we engage with the world, has already been explored in the context of renal colic. In chronic illness, however, this bodily trust is often replaced by what Havi Carel terms bodily doubt. In her book on the phenomenology of illness, Carel describes bodily doubt as a fundamental disruption of *being-in-the-world*, where the body ceases to recede into the background and instead becomes an intrusive object of attention. She writes “it is the loss of a certainty that has hitherto not been disturbed” (2016, p. 94). This shift can precipitate feelings of isolation, detachment, anxiety, and uncertainty. In chronic conditions, this leads to a breakdown in the continuity of daily life: Husserl’s “I can” is increasingly replaced by an “I cannot”, and the horizon of future possibilities narrows.

Patients with chronic kidney stone disease often seem to exhibit this bodily doubt, wherein the illness continually disrupts their trust in the body. This uncertainty manifests as anxiety and hesitation surrounding daily activities, especially those involving work, travel, and the possibility of (unplanned) hospital admissions (Penniston and Nakada 2013; Ragab et al. 2020; Raja, Wood & Joshi 2020). The fear of recurrence and the unpredictability of symptoms forces patients to give up spontaneity. Actions that were once performed seamlessly and pre-reflectively now require deliberate planning. For some, even basic activities like grocery shopping or walking long distances become clouded with worry or anxiety (Raja, Wood and Joshi 2020). As Carel notes, the body in illness becomes difficult to trust: it is no longer an enabler of actions, but a source of interruption or constraints (Carel 2016, pp. 86–105). This doubt is further intensified in those with residual fragments, as “in bodily doubt, we may even worry about aspects of our body that are normally invisible (e.g. liver function) and alter our behavior accordingly” (Carel 2016, p. 100).

In addition to bodily doubt, Merleau-Ponty’s phenomenology offers further insight into how illness undermines the projection of future possibilities. He explains that the *intentional arc*, which “creates the unity of the senses, the unity of the senses with intelligence, and the unity of sensitivity and motricity”, ‘goes limp’ in illness” (Merleau-Ponty 1962/2012, p. 137). This description may capture the experience of chronic kidney stone disease as well, where past experiences of pain and disruption become embedded into the patient’s current existential orientation, thereby constraining the imagination and pursuit of future actions. While in health, the body mediates between present experiences and future goals, illness arrests this synthesis: pain, discomfort and the unpredictability of recurrence suspend meaningful action and force a withdrawal from the world. As Carel puts it, illness causes a shift “as an intentional subject to experiencing oneself as a material object” (2016, p. 99). Toombs similarly observes that in chronic illness, patients often live in constant fear of recurrence, regardless of temporality^5^—a fear that realigns with the unpredictable recurrent nature of renal colic (1988).

Thirdly, chronic kidney stone disease can be associated with depersonalization. Geniusas, drawing on Edmund Pellegrino and David Thomasma, explains that “chronic pain is lived not merely as an assault on our bodies, but as an assault on our personalities” (2022, p. 148). He argues that pain disturbs the relationship between the lived-body and the self, leading to feelings of betrayal by the body, and a loss of the personalistic attitude (Geniusas 2022, pp. 142–163). Pain also robs individuals of the possibility of future actions, which disrupts their sense of agency and diminishes self-confidence and self-reliance. Patients with chronic pain often experience social isolation, as others struggle to understand their pain, and patients themselves may become increasingly dependent. This sense of isolation and misunderstanding can contribute to fragmentation of the self.

According to Geniusas, this may culminate in depersonalization: a shift away from the personalistic attitude to a dissociative experience characterized by detachment from oneself. While feelings of disconnection from others and social isolation are distinct phenomena, they often coexist with depersonalization in chronic pain conditions. Similar experiences are described in kidney stone disease, where patients express feelings of disconnection from others, stating that “no one understand what I [the patient] feel” (Ayyad and Ayaad 2023, p. 5, Raja, Wood & Joshi 2020). This complex interplay of social disconnection and self-detachment may contribute to the destruction of the patient’s sense of self, potentially leading to dissociative symptoms and depersonalization.^6^

A fourth aspect that warrants attention in chronic kidney stone disease is psychologization. Geniusas argues that in cases of persistent somatic pain, psychic complaints often emerge as another dimension of suffering (2022, pp. 164–187). As established in earlier sections, the relationship between body, mind and person is phenomenologically integrated—not composed of clearly distinct or separable parts. The psyche and the soma are fused; thus, psychic states can turn “into a metaphor of somatic feelings”, and vice versa (Geniusas 2022 p. 167). This entanglement becomes especially pronounced when pain is chronic and unresolved (Coninx 2024). In such cases, psychologization may be understood as a response to the difficulty of articulating the lived experience of suffering and the existential conflict between the person and her now-altered life-world. Often, it is an indirect mode of expression, in which the sufferer has become unable to communicate the depth of their experience directly (Geniusas 2022, p. 181).

Just as psychologization is a sophisticated existential response to the perceived senseless the life-world, somatization can be understood as a parallel phenomenon. In somatization, social and personal distress within an altered or incoherent life-world is expressed somatically (Geniusas 2022, pp. 164–187; Coninx 2023; Coninx 2024). Both responses are often hidden from conscious awareness, making them difficult to identify, even by the sufferer themselves (Coninx 2023). We propose that this indirect somatic expression may also occur in a subset of chronic sufferers of kidney stone disease. In these cases, pain may persist despite successful clinical intervention, and psychological distress may intensify. It is as if the kidney stones have been incorporated into body schema, not simply as physical objects, but as existential symbols of suffering. In such instances, surgical removal or medical treatment alone cannot resolve the condition, because the stones now signify something more than their material presence. For these complex, indirect expressions of distress, a different therapeutic approach may be required—one that addresses the broader existential or phenomenological dimensions. Before discussing such approaches, however, we turn to the final aspect of chronic kidney stone disease: *unhomelike being-in-the-world.*

Ordinarily we experience the world as familiar, where we are *being-at-home* in the world, as already introduced previously. However, Heideggerian phenomenology reminds us that this familiarity is never total. Because we can never fully ‘know’ or control the world, and because our existence is inherently shared with others, the world also retains a persistent ‘otherness’, that can render it alien or ‘uncanny’ (Heidegger 1962/2001, pp. 228–235). This alien dimension of experience, when brought to the foreground, such as in anxiety, results in what Heidegger terms unhomelike being-in-the-world (*unheimlichkeit*). In everyday life, this *unhomelike being-in-the-world* tends to remain hidden beneath the more dominating experience of homeliness, as routines and bodily fluency cover up the existential strangeness of existence. However, as Frederik Sveneaus argues, chronic illness, due to its focus on the body and its limitations, can rupture this balance, revealing an unobtrusive *unhomelike being-in-the-world*. Health is then best understood as “a being at home that keeps the not being at home in the world from becoming apparent” (Svenaeus 2001, p. 94).

In chronic kidney stone disease, the persistent disruption of the pre-reflective, unified lived-body, combined with the inability to project and realize future actions—can culminate in alienation, not only from the body, but from the world itself. In such cases, *unhomelike being-in-the-world* overtakes the more fundamental, tacit, homelike being-in-the-world. As Frederik Sveneaus articulates, the unhomelikeness of illness does not merely represent a subjective mood or passing discomfort, rather, it manifests as “a certain form of senselessness, an attunement of, for instance, disorientedness, helplessness, resistance, and despair” (Svenaeus 2001, p. 102). In this view, illness transcends affective states and becomes a total “mode of understanding”, a way of being-in-the-world that is significantly altered (Svenaeus 2000c, p. 10). This reconceptualization of illness may pave the way for new insights into how chronic kidney stone disease ought to be understood and approached: not merely as a medical problem, but as existential disruptions requiring more holistic forms of care, as will be explored in the following section.

## Clinical phenomenology

Now that we have completed our phenomenological analysis of kidney stone disease, we can shift focus to its clinical and scientific implications. The primary goal is to enhance and complement the biomedical perspective, rather than criticize or replace it.

Contemporary care for kidney stone disease continues to advance, and scientists and physicians are increasingly concerned with aspects influencing patient experience. Recently, there has been growing attention to the role of residual fragments and the potential to remove these fragments during the same operative session to reduce the patient’s medical and experiential burden (Kingma et al. 2024; Cracco and Scoffone 2020). Additionally, tubeless procedures, which do not require stents, are becoming more common due to recent breakthroughs (Amer et al. 2012). Modern techniques now also enable intraoperative imaging during sedation, eliminating the need for patients to directly experience imaging and improving operative outcomes (Roemeling et al. 2025; Suijker et al. 2025). Furthermore, metaphylaxis and lifestyle adjustments are increasingly approached in a multidisciplinary manner, often with the support of paramedics who are well-trained in more holistic approaches (Balawender et al. 2024). Patients are also becoming more engaged in treatment decisions and research design (Streeper et al. 2016; Sacristán et al. 2016). However, while these aspects of patient-centered care reflect a focus on political equality and the optimalization of biomedical care, they often lack phenomenological depth. The focus is primarily on improving clinical outcomes, rather than on the deeper evaluation of the patient’s lived experience and lifeworld (Galvin & Todres 2013).

In the phenomenological literature, several proposals have been put forward for integrating phenomenology into clinical practice. Kay Toombs highlights the different perspectives of physicians and patients, advocating for a shift from focusing on the objective body (*Körper*) to understanding the body as lived (*Leib*) through empathy. She emphasizes the importance of empathic listening and the physician imagining themselves in the patient’s situation (Toombs 2001). Havi Carel, on the other hand, suggests a phenomenological toolkit, wherein patients can explore and articulate their illness phenomenologically during a workshop (Carel 2012). Meanwhile, Frederik Svenaeus proposes that clinical medicine serves the function of guiding patients from a state of unhomelikeness back to a sense of *homelike being-in-the-world*. This can be achieved by paying attention to the patient’s life-world an assisting them in adapting to their new reality (Svenaeus 2011).

What stands out in this debate is that phenomenology reveals there is no clear divide between the psyche and the soma. The person, as the subject of pain and illness, expressing lived bodiliness, should influence how we approach these issues in clinical practice. As we have observed, especially in chronic conditions, there is a tendency for both psychologization and somatization to emerge as forms of indirect expression. Saulius Geniusas concludes that “chronic pain, which derives from organic causes, is never only organic” (2022, p. 154) and that since “chronic pain is not reducible to its origin, so its treatment cannot be reduced to its origin’s treatment. If one agrees that responses to pain—bodily, emotive, and cognitive—are part of the very experience of pain, then one also has to concede that chronic pain is never purely physiological or purely psychological” (2022, p. 155). This also aligns with the findings of Sabrina Coninx and Peter Stilwell, who adopt an enactive approach, where “there is no clear pathology to pinpoint and treat” in chronic pain (2021, p. 7854).

In order for physicians to effectively address these issues, it is essential to recognize this interconnectedness of disease, especially in the case of chronic conditions. In kidney stone disease, for example, it is important to understand that pain and illness provoke bodily, emotive and cognitive responses that cannot be solely traced back to the original pathophysiological cause. As a result, one must “supplement curing with healing” (Geniusas 2022, p. 156). Geniusas advocates for a meaningful dialogue, which is rooted in attentive listening, to bridge the ontological gap between the physician, who typically adopts a naturalistic perspective, and the patient, who views situation through the *personalistic attitude* (Geniusas 2022, pp. 154–163). This form of listening helps reveal the patient’s relationship to pain, their connections to others, their future aspirations, and their own personal history. It enables us to explore the patient’s position within their life-world.

Physicians are entrusted with a distinctive role: patients invite them into their life-world, granting access to the most intimate dimensions of their existence. As outlined above, experiences of pain, illness, and care profoundly affect the four fundamental dimensions of the patient’s being: embodiment, personhood, relationality to the world, and connection with others. Attending to these existential structures is not merely a clinical consideration but an ethical imperative, as medical practice is, at its core, concerned with human existence itself.

Following this obligation, and building upon our theoretical phenomenological framework, we can offer the following seven concrete proposals for urological practice, particularly in the care for patients with (recurrent) kidney stone disease:

(I) In order to give appropriate attention to the personalistic attitude and the patient’s life-world, it is important that physicians develop a clearer understanding of these concepts and their clinical relevance. While medical education has traditionally emphasized the objective analysis of the body (*Körper*), there is increasing recognition of the value of complementing this with phenomenological perspectives. Introducing students to key ideas from phenomenology and narrative medicine—whether through dedicated lectures, seminars, or workshops—could foster greater awareness of the lived experience of illness. Such foundations may help clinicians gradually integrate these dimensions into their everyday practice, enhancing their capacity to respond to patients as whole persons.
(II) The personalistic, habitual, lived body should be considered at all stages of clinical interaction, particularly in relation to experiences of anxiety, pain, and the temporal and habitual disruptions associated with procedures that do not involve general anaesthesia, such as stent placement, imaging, extracorporeal shock wave lithotripsy (ESWL), and metaphylaxis. Maintaining an open and empathetic dialogue with the patient during these procedures is crucial to reduce feelings of alienation and objectification, fostering a sense of connection and understanding throughout the experience.
(III) It is important to explore how disease and pain relate to the four fundamental relations—- personhood, body, others, and the world—and to discuss aspects such as bodily doubt, supportive relationships, and the future projects the patient values. Physicians should assess whether the disease and its management impede these future goals. By addressing these issues, physicians can identify and attend to the unhomelike aspects of the patient’s *being-in-the-world*, guiding them toward a more homelike state where they feel more at peace and integrated within their life-world.
(IV) Building on point three, physicians should recognize that the goals of a clinical encounter often differ between the patient and the physician. While the physician typically focuses on medical goals such as “diagnosis, treatment, and prognosis”, the patient interprets these findings from an existential perspective, seeking “explanation, cure, and prediction” (Toombs 1987, p. 227). Therefore, the physician should tailor their consultation to explicitly address how a particular illness impacts the patient’s life, engaging in a dialogue that acknowledges the personal and existential dimensions of the patient’s experience.
(V) Physicians should remain attentive to the structural and practical constraints that may hinder the adoption of a personalistic approach. These may include limited familiarity with phenomenological perspectives, challenges in shifting from a naturalistic to a personalistic attitude—particularly during complex procedures that demand sustained technical focus—as well as time constraints inherent in clinical settings. Acknowledging these limitations transparently in communication with patients can itself foster trust. When appropriate, patients may be referred to colleagues or professionals, such as clinical ethicists or psychologists, who are better positioned to engage with the personalistic dimensions of care, to ensure that these aspects of the patient’s experience are adequately addressed.
(VI) Building on point five, patients with chronic diseases often experience feelings of alienation and isolation. These patients may struggle to share their life-world with relatives or their physician, which highlights the importance of facilitating interactions between patients. Engaging in patient organizations, online fora or more specialized phenomenological workshops, as suggested by Carel (2012), provides a valuable opportunity for patients to share their experiences, reduce feelings of isolation, and foster a sense of connection with others who understand their challenges. Additionally, generative artificial intelligence may also serve as a complementary tool to support these efforts (Pani et al. 2024)
(VII) Finally, in chronic kidney stone disease, it is important to recognize that while the origin of the complaints may appear purely physical, symptoms are often not reducible to their origin. As a result, conventional treatment may be less effective. In such cases, a multidisciplinary approach—one that also addresses indirect psychologization and somatization—can be more successful in helping patients move toward a more homelike state, focusing on guidance on how to interact with the altered life-world, rather than focusing on a complete cure. This holistic approach seems better equipped to establish tranquility while being chronically ill, considering both the physical and existential aspects of the patient's experience.

## Conclusion

This paper has presented the first comprehensive phenomenological analysis of kidney stone disease, examining how the condition disrupts the lived experience of the body, personhood, temporality, and one’s relationship to the world. By drawing on classical phenomenological concepts, such as the lived body (*Leib*), the intentional arc, and *unhomelike being-in-the-world*, we have shown that kidney stone disease, particularly in its recurrent form, is not merely a biomedical problem, but an existential disruption.

While modern urological treatment continues to advance in technical sophistication, it often remains grounded in a naturalistic framework that abstracts from the patient's lived world. The seven practical recommendations we have outlined aim to integrate phenomenological insight into urological practice, thereby fostering a more holistic and ethically grounded approach to care. By attending to the lived reality of patients with kidney stone disease, especially those facing recurrent or chronic manifestations, clinicians can better support their patients not only in managing disease but in reclaiming a meaningful place within their disrupted life-world.

## Footnotes

1 In phenomenological literature, the term ‘disease’ is typically used to describe the phenomenon from a naturalistic, scientific perspective, while ‘illness’ is referred to as the experience of disease, which aligns more closely with the phenomenological viewpoint. However, we consider such distinctions to be unhelpful in phenomenology, as they often result in an artificial separation between the bodily and the phenomenal (see also Geniusas 2022, pp. 154–156).

2 Recently, there has been a growing trend of labelling qualitative interview studies and (auto)biographies ‘phenomenological’, yet the philosophical methodology in these works is often superficial or entirely absent. To avoid confusion, it is important to emphasize that phenomenology is, first and foremost, a philosophical method that demands a careful and rigorous approach.

3 A valuable historical article recounts Montaigne’s experiences with passing kidney stones during his travels, with at least 26 instances of stone passage documented throughout his journeys (Bisaccia et al. 2013).

4 This view is supported by Sveneaus’ conceptualization of the body as a set of tools in the Heideggerian sense, where these tools should not be seen as external objects, but as belonging “to the projective power of the self and consequently tied to the most intimate and irreplaceable parts of the being-in-the-world of the person” (2000b, p. 134).

5 Toombs explains that the fear of recurrence might actually increase rather than decrease over time. She quotes: “It’s two years since I had an attack of my illness. It’s about time for another” (1988, p. 213), which reflects how the anticipation of recurrence can become a persistent concern for individuals. This also resonates deeply with recurrent kidney stone disease.

6 In his work, Geniusas also discusses the possibility of pain being a repersonalizing experience, where patient responses to pain can be understood as a process of reestablishing the relations that pain has disrupted. This conceptualization highlights how pain, rather than solely alienating the individual, can also offer an opportunity for re-engagement with oneself and one's life-world (2022, pp. 150–154).
